# Synthesis, crystal structure and Hirshfeld surface analysis of 5-[2-(di­cyano­methyl­idene)hydrazin-1-yl]-2,4,6-tri­iodo­isophthalic acid ethanol monosolvate

**DOI:** 10.1107/S205698902300676X

**Published:** 2023-08-04

**Authors:** Fargana S. Aliyeva, Gunay Z. Mammadova, Mehmet Akkurt, Sevim Türktekin Çelikesir, Ajaya Bhattarai

**Affiliations:** a Excellence Center, Baku State University, Z. Xalilov Str. 23, Az 1148 Baku, Azerbaijan; b Organic Chemistry Department, Baku State University, Z. Xalilov Str. 23, Az 1148 Baku, Azerbaijan; c Department of Physics, Faculty of Sciences, Erciyes University, 38039 Kayseri, Türkiye; d Department of Physics, Faculty of Science, Erciyes University, 38039 Kayseri, Türkiye; e Department of Chemistry, M.M.A.M.C. (Tribhuvan University), Biratnagar, Nepal; University of Neuchâtel, Switzerland

**Keywords:** crystal structure, hydrogen bonds, three dimensional network, C—I⋯π inter­actions, Hirshfeld surface analysis

## Abstract

The title compound, C_11_H_3_I_3_N_4_O_4_·C_2_H_6_O, crystallizes in the triclinic *P*  space group with one independent mol­ecule and one ethanol solvent mol­ecule in the asymmetric unit. The benzene ring and the methyl­carbonohydrazonoyl dicyanide group of the main mol­ecule makes a dihedral angle of 57.91 (16)°. In the crystal, O—H⋯O and N—H⋯O hydrogen bonds link pairs of mol­ecules, forming dimers with *R*_2_^2^(14) motifs. These dimers are connected by O—H⋯O hydrogen bonds into chains along the *a-*axis direction, forming *R*_2_^2^(16) ring motifs. Further O—H⋯O inter­actions involving the ethanol solvent mol­ecule connect the chains into a three-dimensional network. In addition, C—I⋯π inter­actions are observed. The inter­molecular inter­actions in the crystal structure were qu­anti­fied and analysed using Hirshfeld surface analysis.

## Abstract 2

In the crystal, pairs of mol­ecules are linked by O—H⋯O and N—H⋯O hydrogen bonds forming dimers with 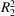 (14) motifs. These dimers are connected by O—H⋯O hydrogen bonds into chains along the *a-*axis direction, forming 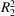 (16) ring motifs. Further O—H⋯O inter­actions involving the ethanol solvent mol­ecule connect the chains into a three-dimensional network.

## 1. Chemical context

Aryl­hydrazones of active methyl­ene compounds (AHAMC) have been extensively employed as ligands and precursors for the synthesis of coordination, organic or supra­molecular compounds (Gurbanov *et al.*, 2020*a* ▸,*b* ▸; Kopylovich *et al.*, 2011 ▸). Besides their biological significance (Martins *et al.*, 2017 ▸), the transition-metal complexes of AHAMC ligands have been found to possess a wide variety of functional properties, and have applications as catalysts, supra­molecular building blocks and analytical reagents (Mahmudov *et al.*, 2010 ▸, 2012 ▸, 2015 ▸). By the functionalization of the active methyl­ene fragment (acetyl­acetone or barbituric acid) or the aromatic moiety (2,4,6-tri­iodo­isophthalic acid) of the AHAMC mol­ecules, the catalytic properties of their metal complexes can be improved in the nitro­aldol reaction between aldehydes and nitro­ethane (Gurbanov *et al.*, 2022 ▸). On the other hand, non-covalent inter­actions such as hydrogen, halogen and chalcogen bonds as well as π-inter­actions can be employed in the synthesis, catalysis and design of materials (Abdelhamid *et al.*, 2011 ▸; Khalilov *et al.*, 2021 ▸; Ma *et al.*, 2021 ▸; Mahmudov *et al.*, 2022 ▸). As well as hydrogen bonds, the cooperation of different weak bonds can act as a driving force for controlling supra­molecular networks (Polyanskii *et al.*, 2019 ▸; Safarova *et al.*, 2019 ▸; Shikhaliyev *et al.*, 2019 ▸; Zubkov *et al.*, 2018 ▸). Similarly to Schiff base complexes (Mahmoudi *et al.*, 2017*a* ▸,*b* ▸, 2019 ▸), the functional groups can be involved in various types of inter­molecular inter­actions in metal complexes of aryl­hydrazone ligands. We have synthesized a new iodine-substituted AHAMC ligand, 5-[2-(di­cyano­methyl­ene)hydrazin­yl]-2,4,6-tri­iodo­isophthalic acid, and studied the inter­molecular halogen bonds and other types of weak inter­actions in its crystal structure. 
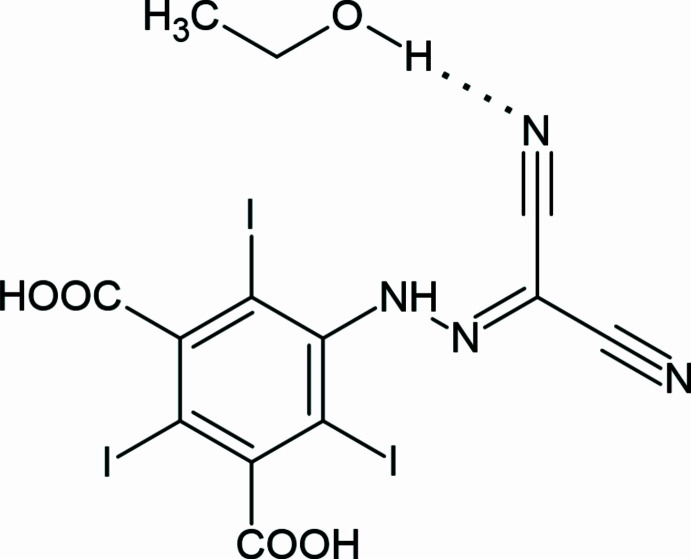


## 2. Structural commentary

**Figure 1:**
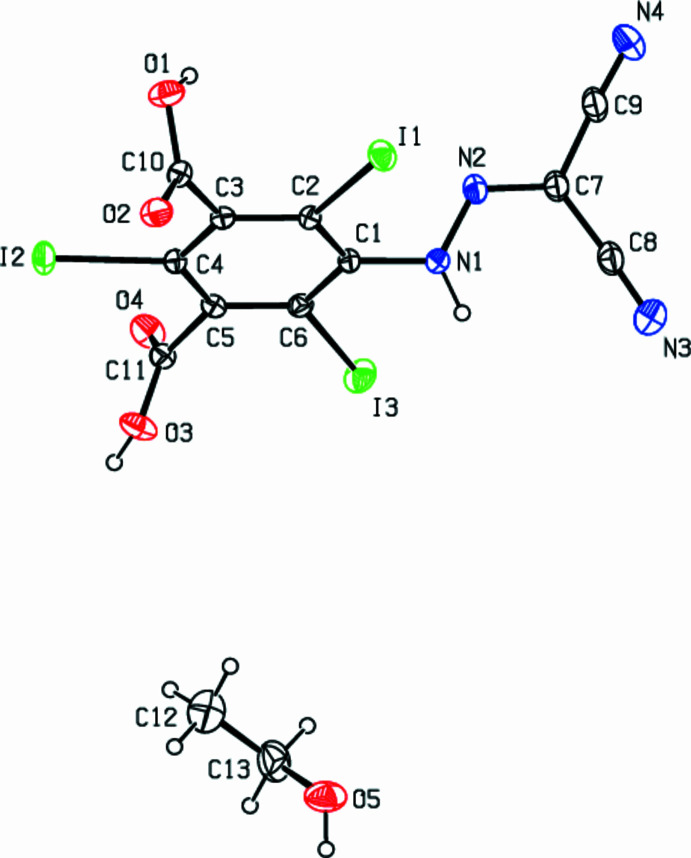
The mol­ecular structure of the title compound, showing the atom labelling and displacement ellipsoids drawn at the 30% probability level.

The title compound (Fig. 1 ▸) crystallizes in the triclinic *P* 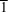 space group with one independent mol­ecule and one ethanol solvent mol­ecule in the asymmetric unit. The benzene ring (C1–C6) and the methyl­carbonohydrazonoyl dicyanide group (N1–N4/C1/C7–C9) of the main mol­ecule makes a dihedral angle of 57.91 (16)°. Geometric parameter values in the mol­ecule are normal and in good agreement with the values in the compounds discussed in the *Database survey* section.

## 3. Supra­molecular features and Hirshfeld surface analysis

**Figure 2:**
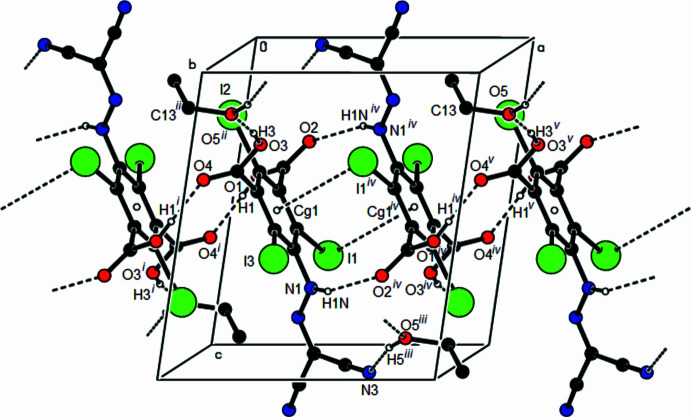
A part view of the mol­ecular packing in the unit cell. N—H⋯O, O—H⋯O hydrogen bonds and C—I⋯π inter­actions are shown as dashed lines. Symmetry codes: (i) −*x*, −*y* + 1, −*z* + 1; (ii) *x* − 1, *y*, *z*; (iii) −*x* + 2, −*y* + 2, −*z* + 1; (iv) −*x* + 1, −*y* + 1, −*z* + 1; (v) *x* + 1, *y*, *z*.

In the crystal of the title compound, pairs of mol­ecules are linked by O—H⋯O and N—H⋯O hydrogen bonds, forming dimers with 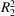 (14) motifs (Bernstein *et al.*, 1995 ▸; Table 1 ▸, Fig. 2 ▸). These dimers are connected along the *a*-axis direction by further O—H⋯O hydrogen bonds, forming 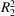 (16) ring motifs. O—H⋯O hydrogen bonds involving the ethanol solvent mol­ecule connect chains into a three-dimensional network. In addition, C—I⋯π inter­actions are also observed [C2—I1⋯*Cg*1(1 − *x*, 1 − *y*, 1 − *z*), 3.8441 (15) Å]. The carbon atoms in the aryl­hydrazone mol­ecule are magnetically non-equivalent as a result of limited rotation around the C—N bond, thus the NH group is locked and becomes ‘*asymmetric*’, which translates into diastereotopic protons and carbons in the title compound.

**Figure 3:**
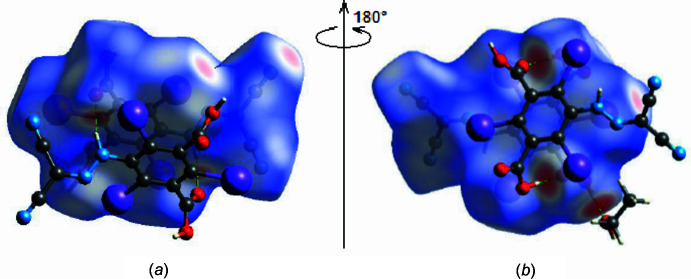
(*a*) Front and (*b*) back sides of the three-dimensional Hirshfeld surface of the title compound mapped over *d*_norm_, with a fixed colour scale of −0.8291 to 1.0734 a.u.

**Figure 4:**
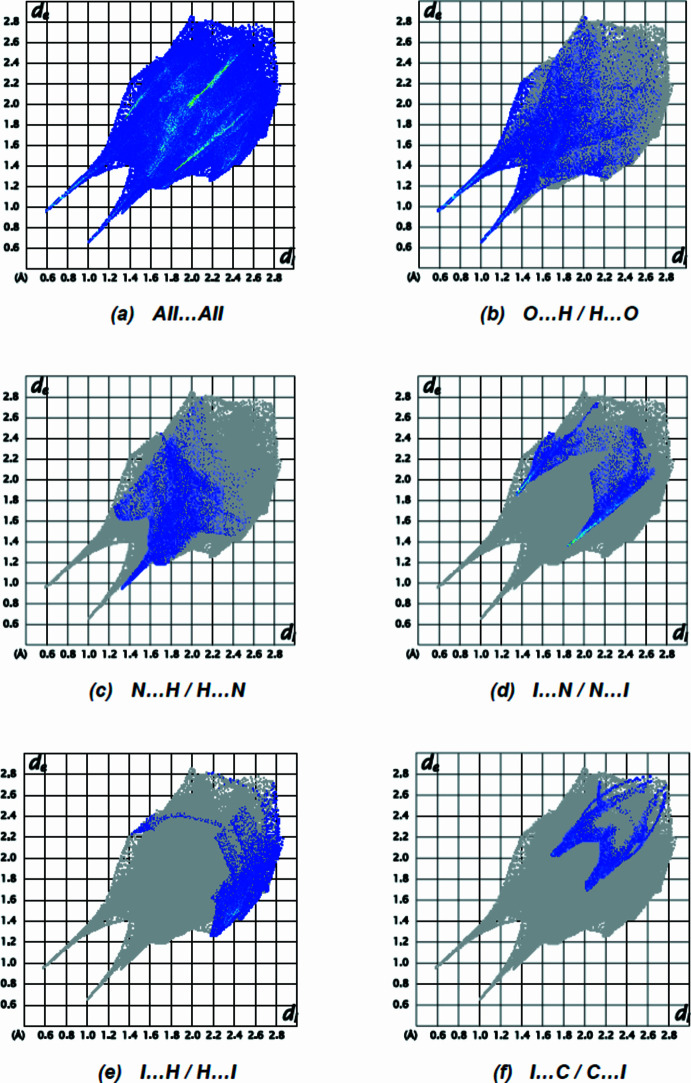
The two-dimensional fingerprint plots of the title compound, showing (*a*) all inter­actions, and delineated into (*b*) O⋯H/H⋯O, (*c*) N⋯H/H⋯N, (*d*) I⋯N/N⋯I, (*e*) I⋯H/H⋯I and (*f*) I⋯C/C⋯I inter­actions. [*d*_e_ and *d*_i_ represent the distances from a point on the Hirshfeld surface to the nearest atoms outside (external) and inside (inter­nal) the surface, respectively.]

In order to present the inter­molecular inter­actions in the crystal structure of the title compound in a visual manner, Hirshfeld surfaces and their associated two-dimensional fingerprint plots were generated using *CrystalExplorer17.5* (Spackman *et al.*, 2021 ▸). The Hirshfeld surface plotted over *d*_norm_ is shown in Fig. 3 ▸, while Fig. 4 ▸ shows the full two-dimensional fingerprint plot and those delineated into the major contacts: O⋯H/H⋯O (23.2%), N⋯H/H⋯N (11.9%), I⋯N/N⋯I (11.9%) and I⋯H/H⋯I (10.7%). Smaller contributions are made by I⋯C/C⋯I (7.7%), C⋯H/H⋯C (6.7%), I⋯O/O⋯I (6.7%), I⋯I (5.4%), C⋯C (4.8%), H⋯H (2.3%), O⋯C/C⋯O(2.3%), N⋯C/C⋯N (2.1%), O⋯N/N⋯O (2.0%), O⋯O (1.4%) and N⋯N (1.0%) inter­actions.

## 4. Database survey

A search of the Cambridge Structural Database (CSD, version 5.43, update June 2022; Groom *et al.*, 2016 ▸) for the *5-amino-2,4,6-tri­iodo­benzene-1,3-di­carb­oxy­lic acid* unit gave four similar structures, *viz.* 5-amino-2,4,6-tri­iodo­isophthalic acid monohydrate (SOGGUR; Beck & Sheldrick, 2008 ▸), 4-(4-pyrid­yl)pyridinium 3-amino-5-carb­oxy-2,4,6-tri­iodo­benzoate–5-amino-2,4,6-tri­iodo­isophthalic acid (1/1) (WADPAU; Zhang *et al.*, 2010 ▸), 5-amino-2,4,6-tri­iodo­isophthalic acid–4,4′-bi­pyri­dine *N*,*N*′-dioxide–water (1/1/1) (UNUDIR; Zhang *et al.*, 2011 ▸) and 5-amino-2,4,6-tri­bromo­isophthalic acid (BOTVUC; Beck *et al.*, 2009 ▸).

In the crystal structure of SOGGUR, mol­ecules are linked by O—H⋯O, N—H⋯O and O—H⋯N hydrogen bonds involving all possible donors and also the water mol­ecule, forming an extensive hydrogen-bond network.

In the ammonium carboxyl­ate–carb­oxy­lic acid co-crystal WADPAU, the carboxyl­ate anion and carb­oxy­lic acid mol­ecule are linked by O—H⋯O and N—H⋯O hydrogen bonds, forming a chain running along the *c*-axis direction of the monoclinic unit cell. The chains are linked by pyridinium and pyridine N—H⋯O hydrogen bonds, generating a layer motif. O—H⋯N and O—H⋯O hydrogen bonds are also observed.

In the crystal of UNUDIR, mol­ecules are linked by O—H⋯O hydrogen bonds into a three-dimensional network. An N—H⋯O inter­action also occurs. One of the amino H atoms is not involved in hydrogen bonding.

In the crystal structure of BOTVUC, mol­ecules are linked into chains by COO—H⋯O bonds, and pairs of chains are connected by additional COO—H⋯O inter­actions. This chain bundle shows stacking inter­actions and weak N—H⋯O hydrogen bonds with adjacent chains.

## 5. Synthesis and crystallization

**Diazo­tization:** 558 mg (1 mmol) of 5-amino-2,4,6-tri­iodo­isophthalic acid were dissolved in 15 mL of water, and the solution was cooled in an ice bath to 273 K, then 69 mg (1 mmol) of NaNO_2_ were added followed by 0.2 mL of HCl, and mixed for 1 h. The temperature of the mixture should not exceed 278 K.

**Azocoupling:** NaOH (40 mg, 1 mmol) was added to a mixture of 1 mmol (66 mg) of malono­nitrile with 5 mL of water. The solution was cooled in an ice bath, and a suspension of 3,5-bis­(meth­oxy­carbon­yl)benzene­diazo­nium chloride (prepared according to the procedure described above) was added in two equal portions under vigorous stirring for 1 h. The precipitate was filtered off, recrystallized from methanol and dried in air. Crystals suitable for X-ray analysis were obtained by slow evaporation of an ethanol solution.

**The title compound:** Yield 79% (based on malono­nitrile), yellow powder soluble in DMSO, methanol, ethanol and DMF. Analysis calculated for C_13_H_9_I_3_N_4_O_5_: C 22.90, H 1.33, N 8.22; found: C 22.87, H 1.30, N 8.18 %. ESI–MS: *m*/*z*: 636.88. IR (KBr): 3123 ν(NH), 2937 ν(NH), 2233 ν(CN) and 1707 ν(C=N) cm^−1. 1^H NMR (300.130 MHz, DMSO-*d*_6_, inter­nal TMS): δ 1.02–1.06 (3H, CH_3_), 3.42–3.47 (2H, CH_2_), 7.25 and 7.32 (2H, COOH) and 11.21 (1H, N—H). ^1^H, in ^13^C{^1^H} NMR (75.468 MHz, DMSO-*d*_6_): δ 18.56 (CH_3_), 56.78 (CH_2_), 85.37, 89.06 and 94.93 (3C–I), 96.80 (C=N), 109.71 and 109.91 (CN), 149.79 and 150.13 (CCOOH), 162.97 (C–NH), 169.48 and 169.79 (C=O).

## 6. Refinement

Crystal data, data collection and structure refinement details are summarized in Table 2 ▸. The hydrogen atoms of the ethanol mol­ecule were placed at idealized positions and refined using a riding model, with *U*_iso_(H) values assigned as 1.2*U*_eq_ or 1.5*U*_eq_(methyl only) of the parent atoms, with C—H distances of 0.97 (methyl­ene) and 0.96 Å (meth­yl). The remaining hydrogen atoms bound to nitro­gen and oxygen were located in difference-Fourier maps and refined with fixed positional thermal displacement parameters and with *U*_iso_(H) values assigned as 1.2*U*_eq_(NH) or 1.5*U*_eq_(OH) of the parent atoms. One reflection, (001), affected by the incident beam-stop was omitted in the final cycles of refinement.

## Supplementary Material

Crystal structure: contains datablock(s) I. DOI: 10.1107/S205698902300676X/tx2072sup1.cif

Structure factors: contains datablock(s) I. DOI: 10.1107/S205698902300676X/tx2072Isup2.hkl

Supporting information file. DOI: 10.1107/S205698902300676X/tx2072Isup3.cml

CCDC reference: 2286318

Additional supporting information:  crystallographic information; 3D view; checkCIF report

## Figures and Tables

**Table 1:** Hydrogen-bond geometry (Å, °)

*D*—H⋯*A*	*D*—H	H⋯*A*	*D*⋯*A*	*D*—H⋯*A*
O1—H1⋯O4^i^	0.85	1.80	2.648 (4)	178
O3—H3⋯O5^ii^	0.85	1.68	2.515 (4)	169
O5—H5⋯N3^iii^	0.85	2.40	3.200 (5)	156
N1—H1*N*⋯O2^iv^	0.92	2.11	2.937 (4)	149

Symmetry codes: (i) 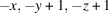 ; (ii) 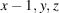 ; (iii) 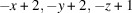 ; (iv) 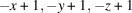 .

**Table 2:** Experimental details

Crystal data	
Chemical formula	C_11_H_3_I_3_N_4_O_4_·C_2_H_6_O
*M*_r_	681.94
Crystal system, space group	Triclinic, *P* 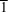
Temperature (K)	296
*a*, *b*, *c* (Å)	9.1499 (3), 9.8771 (3), 12.0440 (4)
α, β, γ (°)	113.512 (1), 95.399 (1), 103.462 (1)
*V* (Å^3^)	949.11 (5)
*Z*	2
Radiation type	Mo *K*α
μ (mm^−1^)	4.97
Crystal size (mm)	0.26 × 0.21 × 0.14

Data collection	
Diffractometer	Bruker D8 Quest PHOTON 100 detector
Absorption correction	Multi-scan (*SADABS*; Krause *et al.*, 2015 ▸)
*T*_min_, *T*_max_	0.325, 0.518
No. of measured, independent and observed [*I* > 2σ(*I*)] reflections	20253, 3755, 3375
*R*_int_	0.025
(sin θ/λ)_max_ (Å^−1^)	0.625

Refinement	
*R*[*F* ^2^ > 2σ(*F* ^2^)], *wR*(*F* ^2^), *S*	0.024, 0.054, 1.22
No. of reflections	3755
No. of parameters	227
H-atom treatment	H-atom parameters constrained
Δρ_max_, Δρ_min_ (e Å^−3^)	0.75, −0.61

Computer programs: *APEX4* and *SAINT* (Bruker, 2008 ▸), *SHELXT2019/1* (Sheldrick, 2015*a* ▸), *SHELXL2019/1* (Sheldrick, 2015*b* ▸), *ORTEP-3 for Windows* (Farrugia, 2012 ▸) and *PLATON* (Spek, 2020 ▸).

## supplementary crystallographic information

### Crystal data

**Table:**

C_11_H_3_I_3_N_4_O_4_·C_2_H_6_O	*Z* = 2
*M**_r_* = 681.94	*F*(000) = 628
Triclinic, *P*1	*D*_x_ = 2.386 Mg m^−^^3^
*a* = 9.1499 (3) Å	Mo *K*α radiation, λ = 0.71073 Å
*b* = 9.8771 (3) Å	Cell parameters from 9873 reflections
*c* = 12.0440 (4) Å	θ = 2.3–26.4°
α = 113.512 (1)°	µ = 4.97 mm^−^^1^
β = 95.399 (1)°	*T* = 296 K
γ = 103.462 (1)°	Prism, orange
*V* = 949.11 (5) Å^3^	0.26 × 0.21 × 0.14 mm

### Data collection

**Table:**

Bruker D8 Quest PHOTON 100 detector diffractometer	3375 reflections with *I* > 2σ(*I*)
φ and ω scans	*R*_int_ = 0.025
Absorption correction: multi-scan (SADABS; Krause *et al.*, 2015)	θ_max_ = 26.4°, θ_min_ = 2.3°
*T*_min_ = 0.325, *T*_max_ = 0.518	*h* = −11→11
20253 measured reflections	*k* = −12→12
3755 independent reflections	*l* = −15→15

### Refinement

**Table:**

Refinement on *F*^2^	Primary atom site location: structure-invariant direct methods
Least-squares matrix: full	Secondary atom site location: difference Fourier map
*R*[*F*^2^ > 2σ(*F*^2^)] = 0.024	Hydrogen site location: mixed
*wR*(*F*^2^) = 0.054	H-atom parameters constrained
*S* = 1.22	*w* = 1/[σ^2^(*F*_o_^2^) + 2.2238*P*] where *P* = (*F*_o_^2^ + 2*F*_c_^2^)/3
3755 reflections	(Δ/σ)_max_ < 0.001
227 parameters	Δρ_max_ = 0.75 e Å^−^^3^
0 restraints	Δρ_min_ = −0.61 e Å^−^^3^

### Special details

**Table:**

Geometry. All esds (except the esd in the dihedral angle between two l.s. planes) are estimated using the full covariance matrix. The cell esds are taken into account individually in the estimation of esds in distances, angles and torsion angles; correlations between esds in cell parameters are only used when they are defined by crystal symmetry. An approximate (isotropic) treatment of cell esds is used for estimating esds involving l.s. planes.

### Fractional atomic coordinates and isotropic or equivalent isotropic displacement parameters (Å^2^)

**Table:**

	*x*	*y*	*z*	*U*_iso_*/*U*_eq_	
I1	0.43837 (3)	0.36375 (3)	0.66860 (2)	0.03614 (8)	
I2	0.00185 (3)	0.27921 (3)	0.22009 (2)	0.03995 (8)	
I3	0.34230 (3)	0.92158 (3)	0.61552 (3)	0.04489 (9)	
O1	0.0961 (3)	0.1098 (3)	0.4380 (3)	0.0419 (6)	
H1	0.063676	0.171314	0.495416	0.063*	
O2	0.2676 (3)	0.1256 (3)	0.3246 (2)	0.0380 (6)	
O3	0.1880 (3)	0.6581 (4)	0.2734 (3)	0.0433 (7)	
H3	0.165558	0.704626	0.231145	0.065*	
O4	0.0089 (3)	0.7033 (4)	0.3831 (3)	0.0438 (7)	
O5	1.0910 (4)	0.7945 (4)	0.1586 (3)	0.0600 (9)	
H5	1.151655	0.834890	0.123379	0.090*	
N1	0.4566 (3)	0.7189 (3)	0.7341 (3)	0.0315 (6)	
H1N	0.532784	0.799315	0.735139	0.038*	
N2	0.4222 (3)	0.7013 (4)	0.8311 (3)	0.0326 (6)	
N3	0.7600 (5)	1.0028 (5)	0.9762 (4)	0.0656 (12)	
N4	0.4455 (6)	0.7440 (5)	1.1278 (4)	0.0656 (12)	
C1	0.3561 (4)	0.6188 (4)	0.6179 (3)	0.0260 (7)	
C2	0.3273 (3)	0.4583 (4)	0.5705 (3)	0.0249 (6)	
C3	0.2269 (4)	0.3621 (4)	0.4560 (3)	0.0262 (7)	
C4	0.1576 (4)	0.4260 (4)	0.3895 (3)	0.0258 (7)	
C5	0.1879 (4)	0.5859 (4)	0.4348 (3)	0.0267 (7)	
C6	0.2875 (4)	0.6808 (4)	0.5487 (3)	0.0262 (7)	
C7	0.5133 (4)	0.7872 (4)	0.9390 (3)	0.0348 (8)	
C8	0.6530 (5)	0.9076 (5)	0.9622 (3)	0.0398 (9)	
C9	0.4713 (5)	0.7606 (5)	1.0428 (4)	0.0437 (9)	
C10	0.1991 (4)	0.1893 (4)	0.4015 (3)	0.0296 (7)	
C11	0.1178 (4)	0.6559 (4)	0.3606 (3)	0.0294 (7)	
C12	0.8376 (7)	0.6247 (7)	0.0688 (6)	0.0785 (17)	
H12A	0.731327	0.619981	0.052211	0.118*	
H12B	0.850672	0.568988	0.116680	0.118*	
H12C	0.869243	0.579198	−0.008073	0.118*	
C13	0.9323 (6)	0.7882 (7)	0.1389 (5)	0.0646 (14)	
H13A	0.915521	0.846280	0.092754	0.078*	
H13B	0.903196	0.833807	0.217868	0.078*	

### Atomic displacement parameters (Å^2^)

**Table:**

	*U*^11^	*U*^22^	*U*^33^	*U*^12^	*U*^13^	*U*^23^
I1	0.04006 (14)	0.03692 (13)	0.03368 (13)	0.01326 (10)	0.00214 (10)	0.01774 (10)
I2	0.03979 (14)	0.03778 (14)	0.02815 (13)	0.00210 (10)	−0.00658 (10)	0.00856 (10)
I3	0.05157 (16)	0.02331 (12)	0.05002 (16)	0.00527 (10)	0.00314 (12)	0.01113 (11)
O1	0.0475 (16)	0.0294 (13)	0.0477 (16)	0.0067 (12)	0.0229 (13)	0.0155 (12)
O2	0.0432 (15)	0.0294 (13)	0.0371 (14)	0.0089 (11)	0.0160 (12)	0.0095 (11)
O3	0.0468 (16)	0.0641 (18)	0.0457 (16)	0.0286 (14)	0.0219 (13)	0.0408 (15)
O4	0.0432 (15)	0.0589 (18)	0.0422 (15)	0.0288 (14)	0.0164 (12)	0.0255 (14)
O5	0.0564 (19)	0.069 (2)	0.081 (2)	0.0197 (17)	0.0171 (17)	0.057 (2)
N1	0.0339 (15)	0.0276 (14)	0.0257 (14)	−0.0020 (12)	0.0005 (12)	0.0119 (12)
N2	0.0340 (16)	0.0339 (16)	0.0241 (14)	0.0074 (13)	0.0016 (12)	0.0092 (12)
N3	0.062 (3)	0.061 (3)	0.054 (2)	−0.010 (2)	−0.002 (2)	0.023 (2)
N4	0.082 (3)	0.072 (3)	0.033 (2)	0.004 (2)	0.0039 (19)	0.024 (2)
C1	0.0239 (15)	0.0290 (16)	0.0196 (15)	0.0023 (13)	0.0028 (12)	0.0087 (13)
C2	0.0204 (15)	0.0278 (16)	0.0241 (15)	0.0027 (12)	0.0015 (12)	0.0120 (13)
C3	0.0233 (15)	0.0291 (16)	0.0259 (16)	0.0038 (13)	0.0060 (13)	0.0137 (14)
C4	0.0261 (16)	0.0253 (16)	0.0209 (15)	0.0028 (13)	0.0038 (12)	0.0079 (13)
C5	0.0283 (16)	0.0293 (16)	0.0229 (15)	0.0068 (13)	0.0076 (13)	0.0122 (13)
C6	0.0281 (16)	0.0217 (15)	0.0269 (16)	0.0042 (13)	0.0066 (13)	0.0102 (13)
C7	0.0380 (19)	0.0336 (18)	0.0267 (17)	0.0075 (15)	0.0001 (15)	0.0102 (15)
C8	0.048 (2)	0.035 (2)	0.0275 (18)	0.0093 (18)	−0.0007 (16)	0.0084 (16)
C9	0.049 (2)	0.044 (2)	0.0283 (19)	0.0081 (18)	−0.0034 (17)	0.0112 (17)
C10	0.0291 (17)	0.0253 (16)	0.0278 (17)	0.0029 (14)	0.0030 (14)	0.0087 (14)
C11	0.0313 (17)	0.0289 (17)	0.0262 (16)	0.0062 (14)	0.0065 (14)	0.0116 (14)
C12	0.073 (4)	0.077 (4)	0.068 (4)	0.007 (3)	0.005 (3)	0.024 (3)
C13	0.063 (3)	0.072 (3)	0.058 (3)	0.016 (3)	−0.009 (2)	0.034 (3)

### Geometric parameters (Å, º)

**Table:**

I1—C2	2.097 (3)	C1—C6	1.397 (5)
I2—C4	2.104 (3)	C1—C2	1.401 (5)
I3—C6	2.097 (3)	C2—C3	1.399 (4)
O1—C10	1.307 (4)	C3—C4	1.392 (5)
O1—H1	0.8499	C3—C10	1.511 (5)
O2—C10	1.214 (4)	C4—C5	1.396 (5)
O3—C11	1.287 (4)	C5—C6	1.390 (5)
O3—H3	0.8500	C5—C11	1.510 (5)
O4—C11	1.205 (4)	C7—C8	1.444 (6)
O5—C13	1.432 (6)	C7—C9	1.445 (6)
O5—H5	0.8500	C12—C13	1.481 (8)
N1—N2	1.303 (4)	C12—H12A	0.9600
N1—C1	1.419 (4)	C12—H12B	0.9600
N1—H1N	0.9222	C12—H12C	0.9600
N2—C7	1.300 (5)	C13—H13A	0.9700
N3—C8	1.134 (6)	C13—H13B	0.9700
N4—C9	1.136 (6)		
			
C10—O1—H1	109.4	C1—C6—I3	119.1 (2)
C11—O3—H3	120.3	N2—C7—C8	124.6 (3)
C13—O5—H5	119.8	N2—C7—C9	117.6 (3)
N2—N1—C1	117.7 (3)	C8—C7—C9	117.8 (3)
N2—N1—H1N	125.8	N3—C8—C7	177.0 (4)
C1—N1—H1N	115.9	N4—C9—C7	176.6 (5)
C7—N2—N1	119.9 (3)	O2—C10—O1	120.9 (3)
C6—C1—C2	119.5 (3)	O2—C10—C3	121.4 (3)
C6—C1—N1	119.8 (3)	O1—C10—C3	117.6 (3)
C2—C1—N1	120.7 (3)	O4—C11—O3	125.5 (3)
C3—C2—C1	119.5 (3)	O4—C11—C5	122.5 (3)
C3—C2—I1	120.3 (2)	O3—C11—C5	112.0 (3)
C1—C2—I1	120.1 (2)	C13—C12—H12A	109.5
C4—C3—C2	120.0 (3)	C13—C12—H12B	109.5
C4—C3—C10	119.7 (3)	H12A—C12—H12B	109.5
C2—C3—C10	120.2 (3)	C13—C12—H12C	109.5
C3—C4—C5	120.9 (3)	H12A—C12—H12C	109.5
C3—C4—I2	119.4 (2)	H12B—C12—H12C	109.5
C5—C4—I2	119.7 (2)	O5—C13—C12	109.1 (5)
C6—C5—C4	118.8 (3)	O5—C13—H13A	109.9
C6—C5—C11	120.1 (3)	C12—C13—H13A	109.9
C4—C5—C11	121.0 (3)	O5—C13—H13B	109.9
C5—C6—C1	121.2 (3)	C12—C13—H13B	109.9
C5—C6—I3	119.6 (2)	H13A—C13—H13B	108.3
			
C1—N1—N2—C7	177.9 (3)	C4—C5—C6—C1	−0.3 (5)
N2—N1—C1—C6	121.9 (3)	C11—C5—C6—C1	−178.8 (3)
N2—N1—C1—C2	−59.3 (4)	C4—C5—C6—I3	177.5 (2)
C6—C1—C2—C3	−1.7 (5)	C11—C5—C6—I3	−1.0 (4)
N1—C1—C2—C3	179.5 (3)	C2—C1—C6—C5	1.6 (5)
C6—C1—C2—I1	176.7 (2)	N1—C1—C6—C5	−179.7 (3)
N1—C1—C2—I1	−2.0 (4)	C2—C1—C6—I3	−176.2 (2)
C1—C2—C3—C4	0.6 (5)	N1—C1—C6—I3	2.5 (4)
I1—C2—C3—C4	−177.8 (2)	N1—N2—C7—C8	1.6 (6)
C1—C2—C3—C10	177.7 (3)	N1—N2—C7—C9	−178.7 (3)
I1—C2—C3—C10	−0.7 (4)	C4—C3—C10—O2	79.7 (4)
C2—C3—C4—C5	0.6 (5)	C2—C3—C10—O2	−97.4 (4)
C10—C3—C4—C5	−176.5 (3)	C4—C3—C10—O1	−98.9 (4)
C2—C3—C4—I2	−178.2 (2)	C2—C3—C10—O1	84.0 (4)
C10—C3—C4—I2	4.7 (4)	C6—C5—C11—O4	−79.6 (5)
C3—C4—C5—C6	−0.8 (5)	C4—C5—C11—O4	102.0 (4)
I2—C4—C5—C6	178.0 (2)	C6—C5—C11—O3	100.3 (4)
C3—C4—C5—C11	177.6 (3)	C4—C5—C11—O3	−78.1 (4)
I2—C4—C5—C11	−3.6 (4)		

### Hydrogen-bond geometry (Å, º)

**Table:**

*D*—H···*A*	*D*—H	H···*A*	*D*···*A*	*D*—H···*A*
O1—H1···O4^i^	0.85	1.80	2.648 (4)	178
O3—H3···O5^ii^	0.85	1.68	2.515 (4)	169
O5—H5···N3^iii^	0.85	2.40	3.200 (5)	156
N1—H1*N*···O2^iv^	0.92	2.11	2.937 (4)	149

Symmetry codes: (i) −*x*, −*y*+1, −*z*+1; (ii) *x*−1, *y*, *z*; (iii) −*x*+2, −*y*+2, −*z*+1; (iv) −*x*+1, −*y*+1, −*z*+1.
